# Sagittal focusing of synchrotron radiation X-rays using a winged crystal

**DOI:** 10.1107/S0909049512049813

**Published:** 2013-01-23

**Authors:** A. Nisawa, Y. Yoneda, G. Ueno, H. Murakami, Y. Okajima, K. Yamamoto, Y. Senba, K. Uesugi, Y. Tanaka, M. Yamamoto, S. Goto, T. Ishikawa

**Affiliations:** a RIKEN Harima Institute SPring-8 Center, Kouto 1-1-1, Sayo, Hyogo 679-5148, Japan; b Kansai Photon Science Institute, Japan Atomic Energy Agency (JAEA), Kouto 1-1-1, Sayo, Hyogo 679-5148, Japan; c Japan Synchrotron Radiation Research Institute (JASRI), Kouto 1-1-1, Sayo, Hyogo 679-5198, Japan

**Keywords:** sagittal focusing, double-crystal monochromator, winged crystal, two-dimensional focusing, crystal bender, SPring-8

## Abstract

A Si(111) winged crystal has been designed to minimize anticlastic bending and improve sagittal focusing efficiency. The crystal was thin with wide stiffening wings. The length-to-width ratio of the crystal was optimized by finite element analysis, and the optimal value was larger than the ‘golden value’. The analysis showed that the slope error owing to anticlastic bending is less than the Darwin width. The X-rays were focused two-dimensionally using the crystal and a tangentially bent mirror. The observed profiles of the focal spot agreed well with the results of a ray-tracing calculation in the energy range from 8 to 17.5 keV. X-ray diffraction measurements with a high signal-to-noise ratio using this focusing system were demonstrated for a small protein crystal.

## Abstract 2

A Si(111) winged crystal has been designed for sagittal focusing of synchrotron radiation X-rays. The results of performance tests at beamlines are reported.

## 1. Introduction

Two-dimensional focusing by combining a bent second crystal in a double-crystal monochromator (Sparks *et al.*, 1980 ▶, 1982 ▶) and a tangential-focusing mirror is an efficient method of increasing the flux density at bending-magnet and wiggler beamlines (*e.g.* Borsboom *et al.*, 1998 ▶; Bilsborrow *et al.*, 2006 ▶; Koshelev *et al.*, 2009 ▶; Nomura & Koyama, 1999 ▶; Yoneda *et al.*, 2005 ▶). Another efficient method is the combination of a double-crystal monochromator with plane–plane crystals and a two-dimensional focusing mirror (Kirkpatrick–Baez mirror, toroidal mirror or tangentially bent cylindrical mirror). The advantages of combining a sagittally bent second crystal and a tangential-focusing mirror over two-dimensional focusing mirror optics are (i) a fixed-height exit over a wide energy range, (ii) a higher flux gain owing to the wider horizontal acceptance, and (iii) the capability of high-energy focusing. In this method the beam size in two-dimensional focusing is sensitive to the shape error of the second crystal from the ideal cylindrical shape owing to anticlastic bending. The anticlastic bending of the second crystal (Sparks *et al.*, 1982 ▶; Kushnir *et al.*, 1993 ▶) leads to a technical difficulty in controlling the sagittal radius of curvature and the parallelism between the first and second crystals within the Darwin width. The anti­clastic bending causes undesirable aberrations and the loss of photon flux in sagittal focusing. Various solutions have been proposed to minimize anticlastic bending, such as by using a ribbed (Borsboom *et al.*, 1998 ▶; Bilsborrow *et al.*, 2006 ▶), hinged or slotted crystal (*e.g.* Sparks *et al.*, 1982 ▶; Kushnir *et al.*, 1993 ▶; Schulze *et al.*, 1998 ▶; Yoneda *et al.*, 2001 ▶; Feng *et al.*, 2008 ▶; Koshelev *et al.*, 2009 ▶) with various types of bend mechanism for bending-magnet, wiggler and undulator beamlines. Since a slotted crystal has a smoother reflective surface than both ribbed and hinged crystals, it is suitable for fine focusing. The magnitude of anticlastic bending depends on the aspect ratio of a rectangular crystal. The anticlastic bending at the center of a crystal is minimized at the ‘golden value’ of 1.42 (Kushnir *et al.*, 1993 ▶), which is the ratio for a rectangular Si(111) focusing crystal with ‘clamped’ or ‘built in’ boundary conditions (Kushnir *et al.*, 1993 ▶; Quintana *et al.*, 1995 ▶).

To achieve two-dimensional fine focusing, one solution is to ensure that the bent crystal maintains a continuous cylindrical shape over a wide area of the crystal. We designed a Si(111) rectangular slotted crystal with thick and wide stiffening wings, the so-called ‘winged crystal’, for use with a four-point crystal bender. The amount of anticlastic bending was analyzed by finite element analysis (FEA). We optimized the aspect ratio (length-to-width ratio) of the winged crystal to minimize anticlastic bending while maintaining a continuous cylindrical shape and parallelism to the first crystal within the Darwin width.

We report the focusing performance using the winged crystal. Preliminary results are also shown for the diffraction of a small protein crystal of hen-egg lysozyme with a high signal-to-noise ratio by matching the beam size to the sample size.

## 2. Sagittal focusing crystal

**Figure 1:**
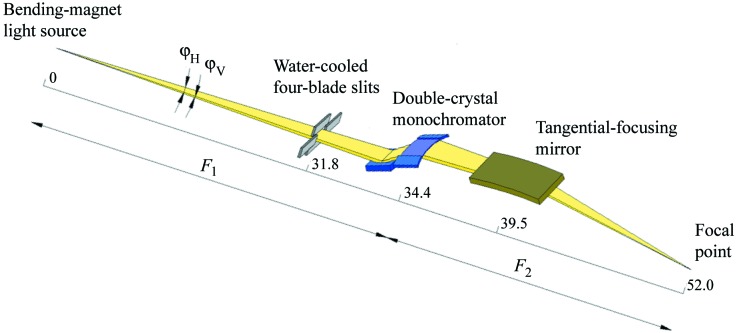
Schematic layout of BL26B1 and BL26B2. ϕ_H_ and ϕ_V_ are the horizontal and vertical acceptance angles of the incident X-rays from the bending-magnet light source, respectively. *F*_1_ and *F*_2_ are the distances from the double-crystal monochromator to the light source and focal point, respectively. The distances between components are presented in meters.

**Figure 2:**
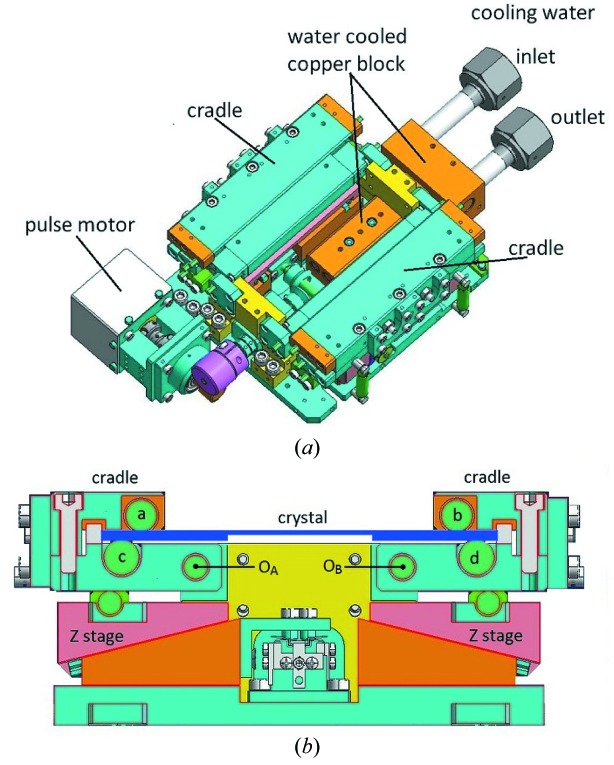
(*a*) Schematic of the four-point crystal bender for the second crystal (Kohzu Precision) and (*b*) the actual bending mechanism when the crystal is flattened. The geometrical condition of this bender is the same as that of a SPring-8 standard bender (Yoneda *et al.*, 2001 ▶). The crystal was clamped with cylindrical rollers (*a*, *b*, *c* and *d*) of the cradles. The effective length of the rollers is 110 mm. The center distance of the upper cylindrical rollers (*a* and *b*) is 80 mm, and that of the lower cylindrical rollers (*c* and *d*) is 90 mm. The bending was performed by rotating the cradles around *O*_*A*_ and *O*_*B*_ owing to the vertical motion of mechanically linked *Z* stages.

**Figure 3:**
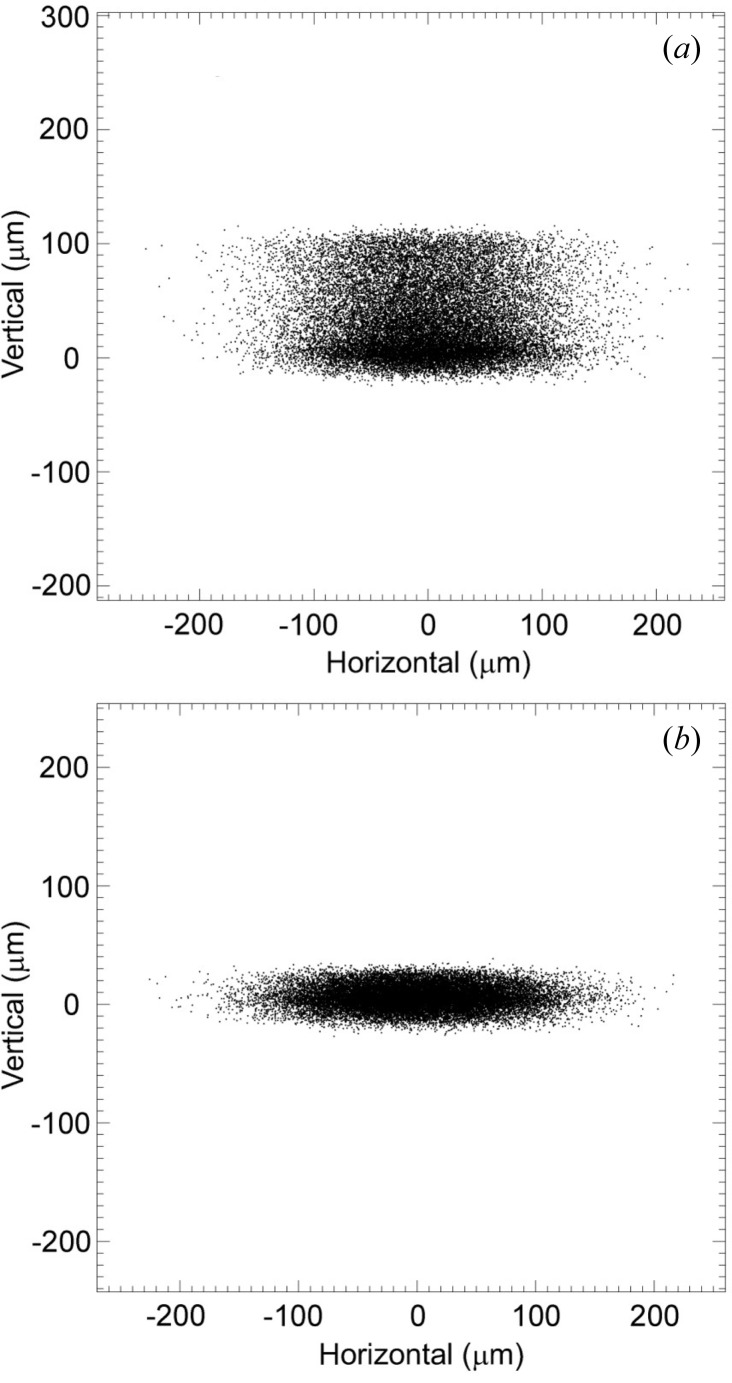
Ray-tracing results for focal beam images at 12.4 keV in the ideal case using the geometry of BL26B1 and BL26B2 (Fig. 1 ▶) with horizontal acceptance angle ϕ_H_ values of (*a*) 1.5 mrad and (*b*) 0.7 mrad. The vertical acceptance of the incident X-rays, ϕ_V_, was set to 30 µrad in both cases. The light source is a 0.679 T bending-magnet source with source electron beam sizes of σ_*x*_ = 106 µm and σ_*y*_ = 13 µm and emittances of ∊_*x*_ = 3.4 × 10^−9^ m rad and ∊_*y*_ = 6.8 × 10^−12^ m rad (Tanaka & Kitamura, 2001 ▶). The sagittal crystal radius of curvature *R*_s_ is 3.71 m. The glancing angle of the tangential-focusing mirror θ_m_ is 3.6 mrad, and the radius of curvature *R*_m_ is 5.27 km.

The characterization of two-dimensional focusing by a bent crystal with a tangential-focusing mirror was carried out using the optical geometry of beamlines BL26B1 and BL26B2 (RIKEN Structural Genomics beamlines I and II) of SPring-8 (Ueno *et al.*, 2006 ▶). Fig. 1 ▶ shows a schematic layout of BL26B1 and BL26B2. The maximum acceptance angles of the double-crystal monochromator, ϕ_H_ and ϕ_V_, are 1.5 mrad and 200 µrad, respectively. Water-cooled four-blade slits and a SPring-8 standard double-crystal monochromator (Uruga *et al.*, 2001 ▶; Yabashi *et al.*, 1999 ▶) are located at a distance of 31.8 m and 34.4 m from the light source, respectively. A directly water-cooled Si(111) crystal (Nisawa *et al.*, 2013 ▶) was used as the first crystal of the double-crystal monochromator. Figs. 2(*a*) and 2(*b*) ▶ show schematic drawings of the four-point crystal bender for the second crystal (Kohzu Precision), which has a higher mechanical reliability on the bending mechanisms against the reactive force of a bent crystal. The rhodium-coated tangential-focusing mirror is located at a distance of 39.5 m from the light source. The glancing angle of the mirror θ_m_ was set to 3.6 mrad. The focal point of the two-dimensional focusing X-ray beam is 52.0 m from the light source. The magnification of sagittal focusing, *M* (= *F*_2_/*F*_1_), is approximately 1/2. By performing ray-tracing calculations for the ideal cylindrical shapes of the crystal and mirror, we found that the horizontal beam width is limited owing to aberration. Fig. 3 ▶ shows the results of the ray-tracing calculations using *SHADOW* (Welnak *et al.*, 1994 ▶) running under *XOP* (*X-ray Oriented Programs*; Sanchez del Rio & Dejus, 1997 ▶, 1998 ▶). The two-dimensional focal beam images become a sharply defined core without a single-sided tail owing to the external curvature of the crystal (Sparks *et al.*, 1980 ▶) when ϕ_H_ is fixed at 0.7 mrad, as shown in Figs. 3(*a*) and 3(*b*) ▶. The optimal horizontal acceptance angle is 0.7 mrad, which reduces the tails to less than 10 µm for energies of 8 to 17.5 keV. The horizontal footprint of the X-ray beam on the second crystal surface is approximately 24 mm for an acceptance angle of 0.7 mrad. We have designed the rectangular slotted crystal based on the dimensions of a SPring-8 standard sagittal crystal (Yoneda *et al.*, 2003 ▶, 2005 ▶) taking into account the results of ray-tracing calculations.

**Figure 4:**
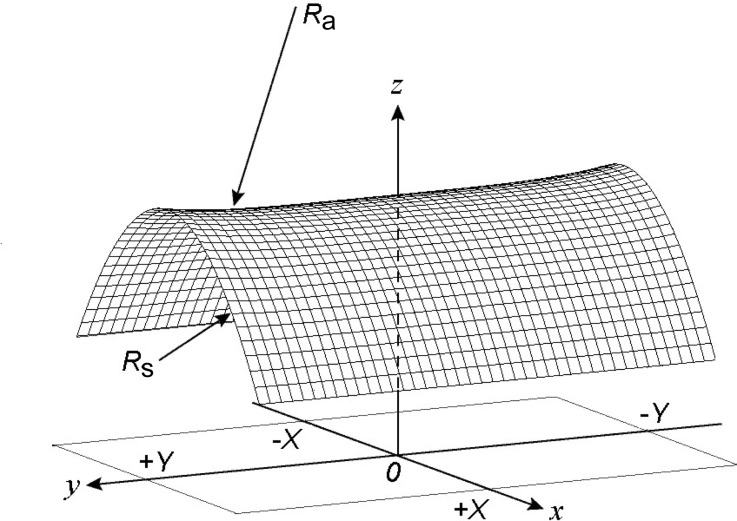
Typical shape of a bent crystal of dimensions 2*X* × 2*Y* subjected to anticlastic bending. *R*_s_ is the sagittal radius and *R*_a_ is the anticlastic radius. The length-to-width ratio (γ) is given by *Y*/*X*, where *X* and *Y* are the half-width and half-length of the crystal, respectively.

We should carefully examine the aspect ratio of the sagittal crystal to avoid anticlastic bending effects. Fig. 4 ▶ shows a schematic of the anticlastic deformation in an isotropic rectangular thin crystal (2*X* × 2*Y*) with a simply supported edge boundary condition; a constant moment is applied along the edges (*y* = ±*Y*). The anticlastic curvature is defined by a function of the crystal’s aspect ratio (length-to-width ratio), γ = *Y*/*X*. Kushnir *et al.* (1993 ▶) have shown that in order to obtain a small curvature at the crystal center point (*x* = *y* = 0) the aspect ratio must be large (γ > 7) or it must be equal to a certain golden value, γ_0_. For a Poisson coefficient of 0.262, the golden value is 2.360. Under clamped edge boundary conditions [*z*(*x*, *y*) = 0 and 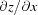 = ‘constant’ at *x* = ±*X*] the golden value decreases to 1.42. However, it is difficult to provide a simply supported edge boundary condition in a four-point bender (Quintana *et al.*, 1995 ▶; Kushnir *et al.*, 1993 ▶). We have determined an optimum design of the crystal by FEA using the *ANSYS* program (ANSYS, 2007 ▶) with the physically controllable clamped crystal boundary condition in a four-point crystal bender.

**Figure 5:**
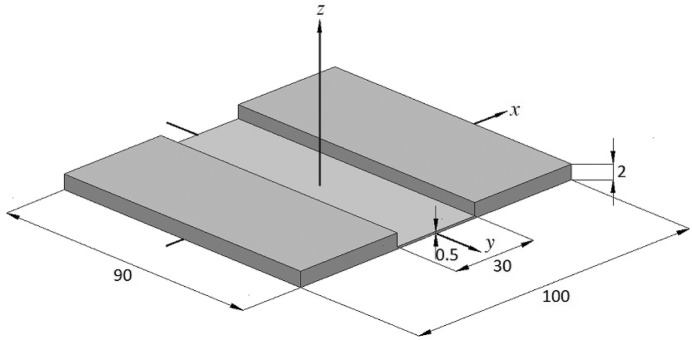
Schematic of the Si(111) rectangular slotted second crystal (winged crystal). The dimensions of the crystal are given in millimeters. The geometry of the slotted area corresponds to that shown in Fig. 4 ▶, *i.e.* 2*X* = 30 mm, 2*Y* = 90 mm and γ = 3.0.

The final design of the winged crystal is shown in Fig. 5 ▶. The crystal has thick and wide wings, which improve its rigidity, similarly to the stiffening effect of ribs for a ribbed crystal. The dimensions of the crystal were fixed to values required for an advanced four-point crystal bender, as shown in Fig. 2 ▶. The aspect ratio of a SPring-8 standard sagittal crystal (Yoneda *et al.*, 2003 ▶, 2005 ▶) is γ = 1.435, corresponding to a width 2*X* of 62.7 mm in the thin region. The optimized width obtained by the analysis is 30 mm (Fig. 5 ▶), corresponding to a ratio of γ = 3 and an acceptance angle of ϕ_H_ = 0.9 mrad. This is larger than the optimum value for two-dimensional focusing of ϕ_H_ = 0.7 mrad.

**Figure 6:**
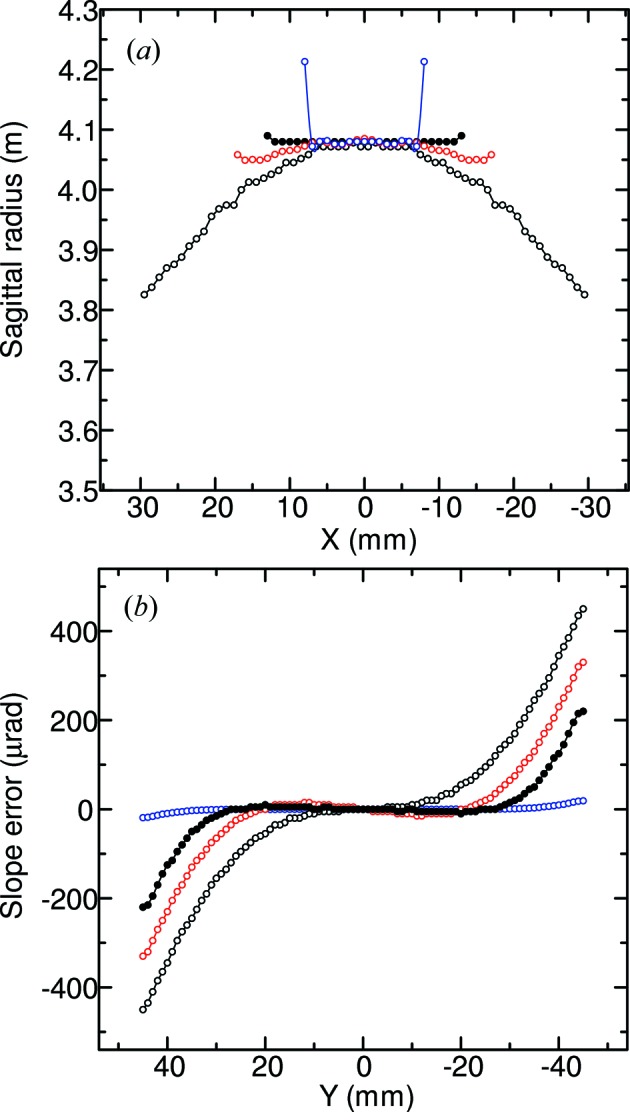
FEA results for crystals with γ = 1.435 (black open circles), 2 (red open circles), 3 (black filled circles) and 4 (blue open circles); (*a*) sagittal radius distributions along the sagittal *X*-direction for *Y* = 0, and (*b*) slope error distributions along the meridional *Y*-direction for *X* = 0.

Figs. 6(*a*) and 6(*b*) ▶ show the FEA results for the sagittal radius (*R*_s_) distributions of the slotted area across the meridional center (*Y* = 0) and the slope error distributions along the meridional centerline owing to the anticlastic bending, respectively. FEA simulations were performed with clamped conditions in a four-point crystal bender (see Figs. 5 ▶ and 2 ▶) fixed at *y*(*x*, *z*) = *y*(±40, 0), and with the applied uniform displacement *UZ* at *y*(*x*, *z*) = *y*(±45, 2). The Poisson coefficient for the Si(111) plane and the sagittal radius were set to 0.262 and 4.1 m, respectively. The FEA results of the crystals were presented with aspect ratios of 1.435, 2, 3 and 4. The widths of 2*X* for those aspect ratios were 62.7 mm, 45 mm, 30 mm and 22.5 mm, respectively. The applied uniform displacement *UZ* values for the crystals were −40 µm, −30 µm, −20 µm and −17 µm, respectively. From these results it was clarified that the anticlastic bending effect decreases with increasing aspect ratio γ. The anticlastic bending is almost reduced over a slotted area with γ = 4. This aspect ratio γ is smaller than other reported large aspect ratios of 6 (Schulze *et al.*, 1998 ▶), 6.6 (Bilsborrow *et al.*, 2006 ▶) and 7.6 (Frenkel *et al.*, 1996 ▶). In this case, 2*X* of 22.5 mm is smaller than the 24 mm required for the optimal horizontal acceptance angle of 0.7 mrad for two-dimensional focusing. When we set the aspect ratio γ to 4, 2*X* should be 30 mm to ensure the same optimal horizontal acceptance angle of 0.7 mrad as that of the γ = 3 crystal, and the crystal length 2*Y* should be stretched from 90 mm to 120 mm. Since 2*Y* of 120 mm is larger than the effective length of the rollers (110 mm) in our four-point bender, the stretched γ = 4 crystal is impractical. In practice, the γ = 3 crystal with 2*X* = 30 mm satisfies the requirements for the optimal horizontal acceptance angle of 0.7 mrad. For the winged crystal with γ = 3, the sagittal radius distribution maintains a continuous cylindrical shape with minimal anticlastic bending over a wide central area of 26 mm (2*X*) × 50 mm (2*Y*). In this area the maximum slope error is 5 µrad, indicating that anticlastic bending is negligible. In the same area for the γ = 2 and 1.435 crystals, the maximum slope errors are 20 µrad and 90 µrad, respectively. In addition, the FEA results of the golden value of 1.42 (2*X* = 63.38 mm) were almost the same as those of 1.435 in our analysis. The aspect ratio γ = 3 deviates from Kushnir’s ideal aspect ratio, golden value or γ > 7 to prevent anticlastic bending effects. The FEA results show that the anticlastic bending of the rectangular slotted crystal with aspect ratio γ = 3 can be minimized under our clamping boundary condition. We have determined the aspect ratio γ of the winged crystal to be 3 by ray-tracing calculation and FEA analysis to achieve two-dimensional fine focusing, as shown in Fig. 6 ▶.

## 3. Performance test at synchrotron radiation beamlines

**Figure 7:**
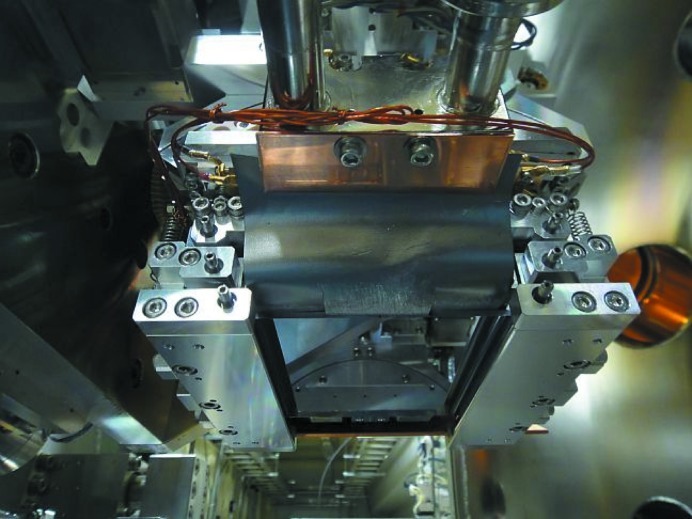
Four-point bender for the winged crystal installed in the double-crystal monochromator as a second crystal.

The Si(111) winged crystal with γ = 3 (Fig. 5 ▶) was manufactured by high-precision machining and chemical etching (Sharan Instruments), and mounted on the second crystal stage with the advanced four-point crystal bender (Kohzu Precision). It was installed in the second crystal stage of the double-crystal monochromator at BL26B1 as shown in Fig. 7 ▶. The bender was cooled by water to prevent heat loading due to Compton scattered X-rays from the first crystal. The winged second crystal was also indirectly cooled by water *via* thin pyrolytic graphite sheets (PGS, Panasonic), which were brought into contact with the bottom surface of the crystal using a liquid gallium–indium (Ga–In) alloy. The horizontal and vertical acceptance angles of the incident X-rays from the bending-magnet light source were set to 0.7 mrad and 30 µrad, respectively, using the water-cooled four-blade slits.

The rocking curves were measured using an ion chamber to assess the effect of the winged crystal on cylindrical bending. The two-dimensional beam profiles at the focal point were observed using an X-ray beam monitor combined with a charge-coupled device (CCD) camera in the energy range from 8 to 17.5 keV, lower than the critical energy of a mirror reflection. The photon flux for several X-ray energies at the focal point was measured using a Si pin-photodiode detector.

## 4. Results and discussion

**Figure 8:**
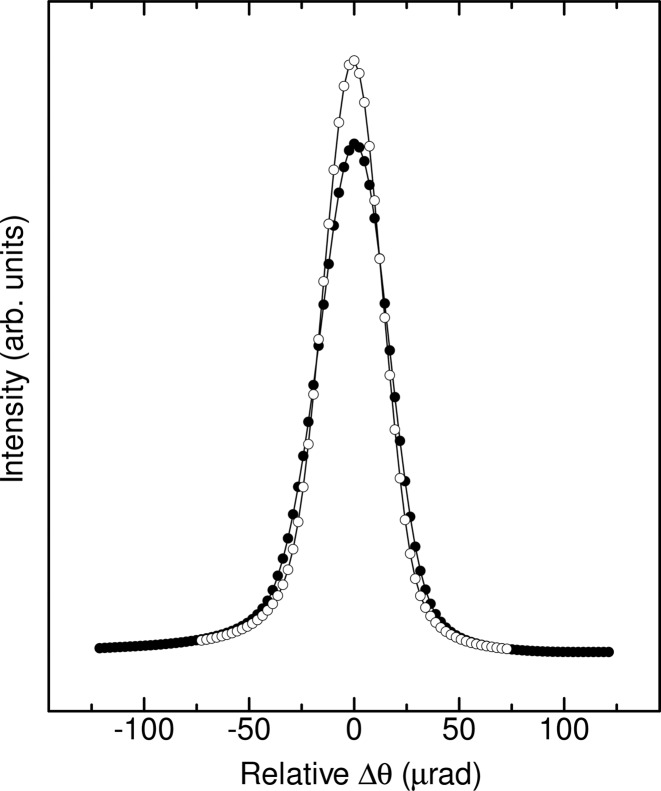
Observed rocking curves for total flux at 12.4 keV using a fully sagittally focused crystal of *R*_s_ = 4 m (filled circles) and a flattened crystal of *R*_s_ = ∞ (open circles). The full widths at half-maximums (FWHMs) of the rocking curves for the fully focused and flattened crystals are 39 and 34 µrad, respectively. The FWHM of the double-crystal rocking curve calculated by using *XOP* is 30 µrad.

Fig. 8 ▶ shows the obtained rocking curves at 12.4 keV for a bent crystal of *R*_s_ = 4 m and for a flattened crystal of *R*_s_ = ∞. *R*_s_ was determined from the amount of vertical motion of the mechanically linked *Z* stages. The FWHM of the flattened crystal of *R*_s_ = ∞ was 4 µrad broader than the FWHM calculated from the double-crystal rocking curve. The beam sizes (vertical × horizontal) at the focal position for *R*_s_ = 4 m and *R*_s_ = ∞ were 1.8 mm × 140 µm and 1.5 mm × 35 mm, respectively. The peak intensity (total photon flux) was approximately 8% lower and the rocking curve was approximately 5 µrad broader for the bent crystal of *R*_s_ = 4 m. The flux density at the sample position with focusing increased approximately 200 times more than that for the flattened crystal of *R*_s_ = ∞. Significant reductions in peak flux and energy resolution due to bending were not observed. The symmetrical rocking-curve profile suggests that the winged crystal was bent cylindrically and that anticlastic bending was minimized.

**Figure 9:**
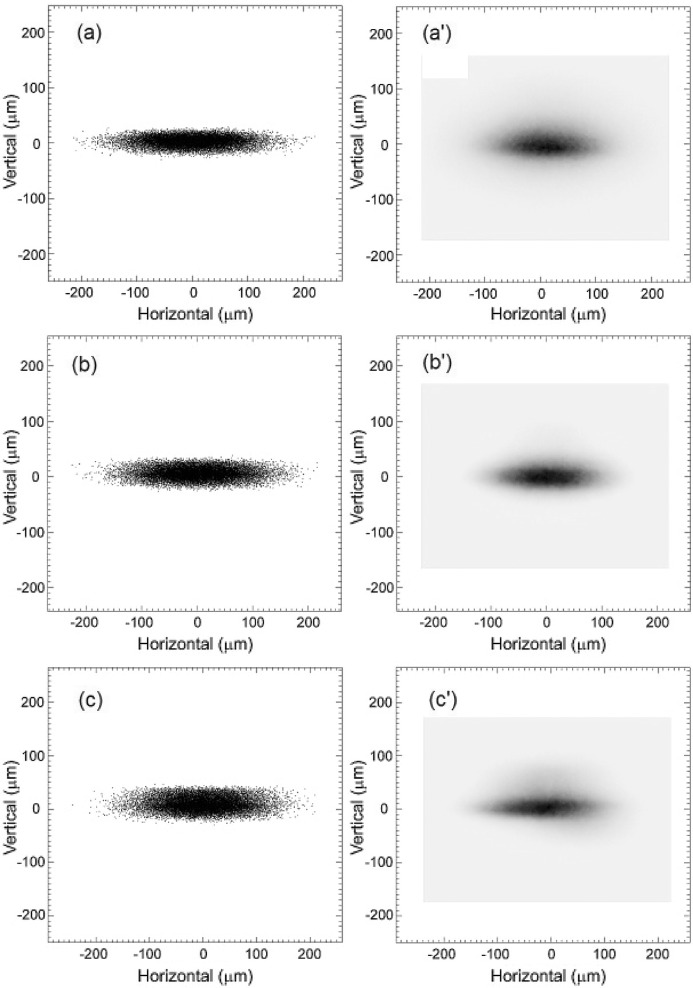
Spatial profiles obtained by two-dimensional focusing. (*a*)–(*c*) Calculated beam images obtained by ray-tracing calculations using *SHADOW* software for energies of 8, 12.4 and 17.5 keV, respectively. (*a*′)–(*c*′) Observed beam profiles at the focal point.

The spatial profiles obtained by two-dimensional focusing with a tangential-focusing mirror were observed in the energy range from 8 to 17.5 keV. Figs. 9(*a*)–9(*c*) ▶ show the calculated two-dimensional focusing beam images obtained by ray-tracing calculations using *SHADOW* software for energies of 8, 12.4 and 17.5 keV, respectively, where (*a*′)–(*c*′) are the corresponding observed spatial profiles at the focal point. The observed spatial profiles have a sharply defined core for each energy. These profiles agree well with the results of the ray-tracing calculations. The dimensions of the focused beams are compared with those in the ray-tracing results in Table 1 ▶. The observed vertical widths of the focusing beams are larger than those in the ray-tracing results. This broadening of vertical widths may be caused by the undesirable slope errors of the first crystal, winged crystal and tangentially bent cylindrical mirror. The slope error of the tangentially bent cylindrical mirror is due to a surface figure error from the ideal cylindrical shape. The slope error of the winged crystal is due to the mounting stress of rollers and the thickness variation of the crystal (Schulze *et al.*, 1998 ▶). At higher energies the thickness variation and anticlastic bending effects of the crystal increase with the decrease in bending radius, and the footprint in the meridional direction on the winged crystal surface becomes large. The effective slope error of the winged crystal becomes slightly larger at higher energies. This slope error reduces the vertical divergence of the output X-ray beam from the double-crystal monochromator, and the observed vertical width of the focusing beam decreases at higher energies.

Table 2 ▶ shows the observed photon fluxes, the ratios of the photon fluxes and the ratios of the focal beam cross sections compared with those for the crystal with γ = 1.435, which were observed at BL26B2 with the same acceptance angles of ϕ_H_ = 0.7 mrad and ϕ_V_ = 30 µrad. It is clear that the improvement factors of the photon flux and the cross section for the winged crystal at the focal position are significantly larger at higher energies, where the sagittal radius is smaller and the footprint in the meridional direction is larger. The higher flux density ratios for the winged crystal are mainly caused by the decrease in beam size at the focal position. A key to achieving a small vertical beam size in the system is to eliminate the vertical smearing that can be seen in Fig. 3 ▶. Since the anticlastic bending of the winged crystal has been minimized for a wide sagittal radius *R*_s_, as shown in Fig. 6 ▶, two-dimensional fine focusing with a tangential-focusing mirror was successfully achieved with a very small loss of photon flux.

**Figure 10:**
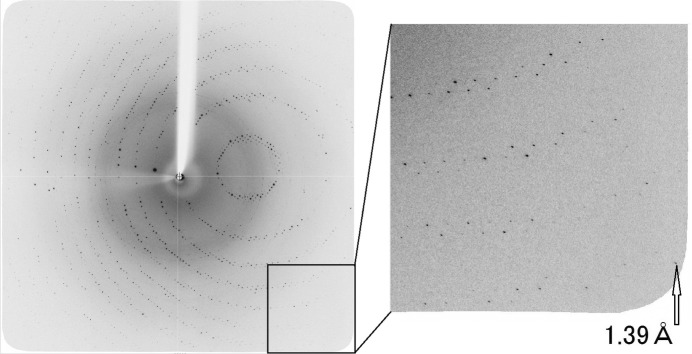
Diffraction image of a protein crystal (hen-egg lysozyme) observed using a CCD area detector (Saturn A200, Rigaku) located 150 mm from the sample. The inset shows the corresponding resolution of the diffraction spot.

The two-dimensional finely focused beam with high photon flux enables the observation of X-ray diffraction data with a high signal-to-noise ratio from small protein crystals, because the diffraction signal is enhanced and the background scattering around the sample is reduced. Fig. 10 ▶ shows an example of an observed diffraction image of a protein crystal sample (hen-egg lysozyme) obtained by the two-dimensional focusing of an X-ray beam with a photon flux of ∼10^11^ photons s^−1^ and a photon energy of 12.4 keV (λ = 1 Å). The crystal size was 70 µm × 100 µm × 80 µm, and the oscillation condition was a 1° rotation with 2 s exposure per frame. The diffraction spots were very clear, and no distortion was observed up to the highest resolution of the detector aperture. The statistics obtained by data processing using the *HKL-2000* program package (Otwinowski & Minor, 1997 ▶) are shown in Table 3 ▶. As a result of the structure analysis by deriving structure factors from the data set shown in Table 3 ▶, a clear electron density map for identifying each amino acid residue in the main chain was obtained.

A comparative measurement was conducted to investigate the improved performance of the monochromator to enhance the weak signals of high-resolution diffraction spots. Two sets of diffraction data at the same exposure time and oscillation width from a lysozyme crystal were obtained with the sagittal and conventional focusing beams. The conventional focusing beam was provided by combining plane–plane crystals and a tangentially bent cylindrical mirror. A comparison of the statistics is shown in Table 4 ▶. The data set obtained with the sagittal focusing beam showed significantly improved *R*-merge and Mean *I*/σ(*I*), particularly in high-resolution shells, owing to the enhanced signal with the sagittally focusing beam.

## 5. Summary

We designed a Si(111) winged crystal to minimize anticlastic bending in sagittal focusing. The crystal had a thin rectangular area at its center, an aspect ratio (γ) of 3, and wide stiffening wings. It also had a uniform sagittal radius and reduced surface slope error due to anticlastic bending compared with a crystal of γ = 1.435. Two-dimensional focusing was achieved with a Si(111) winged crystal and a tangential-focusing mirror without any undesirable aberrations in the energy range from 8 to 17.5 keV. This focusing system can be used to obtain high-quality data with a high signal-to-noise ratio for the diffraction of small protein crystals. Achievements of structural biology research have already been published (*e.g.* Kounosu *et al.*, 2008 ▶; Saino *et al.*, 2011 ▶). The winged crystal proposed here is also applicable to sagittal focusing with a Si(311) crystal monochromator in an adjustable inclined geometry (Uruga *et al.*, 2001 ▶), and performance tests are ongoing.

## Figures and Tables

**Table 1:** Comparison between two-dimensional focusing dimensions obtained by ray-tracing and observation for energies of 8, 12.4 and 17.5 keV *V*_cal_ and *H*_cal_ are the vertical and horizontal dimensions (FWHM), respectively, of focal images obtained by ray-tracing calculations using *SHADOW* software. *V*_obs_ and *H*_obs_ are the observed values.

Energy (keV)	*R*_s_ (m)	*V*_cal_ (µm)	*H*_cal_ (µm)	*V*_obs_ (µm)	*H*_obs_ (µm)
8	6.2	18.6	132.4	44.5	139.5
12.4	4.0	22.5	133.6	40.1	139.4
17.5	2.8	24.0	132.9	35.0	142.2

**Table 2:** Observed photon flux for two-dimensional focusing with energies of 8, 12.4 and 17.5 keV *I*_γ=3_ and *I*_γ=1.435_ indicate the observed photon fluxes for crystals with γ = 3 and 1.435, respectively. *S*_γ=3_ and *S*_γ=1.435_ indicate the observed focal beam cross sections for crystals with γ = 3 and 1.435, respectively. *D*_γ=3_ and *D*_γ=1.435_ indicate the observed flux densities for crystals with γ = 3 and 1.435, respectively.

Energy (keV)	Photon flux (photons s^−1^)	Flux ratio (*I*_γ=3_/*I*_γ=1.435_)	Cross-section ratio (*S*_γ=3_/*S*_γ=1.435_)	Flux density ratio (*D*_γ=3_/*D*_γ=1.435_)
8	4.56 × 10^10^	1.1	0.63	1.75
12.4	1.22 × 10^11^	1.1	0.71	1.55
17.5	9.40 × 10^10^	1.5	0.53	2.83

**Table 3:** Statistics of the diffraction data set for a hen-egg lysozyme crystal Data collection conditions: oscillation range: 0–360° in 1° steps (360 images); exposure time: 2 s per frame. Values in parentheses refer to the highest-resolution shell (1.75–1.69 Å). *R*-merge is the residual among equivalent reflections expressed as Σ|*I* − 〈*I*〉|/Σ〈*I*〉.

Space group	*P*4_3_2_1_2
Cell constants (Å)	*a* = 78.98, *b* = 36.98
Wavelength (Å)	1.00
Resolution (Å)	50.0–1.69 (1.75–1.69)
No. of observations	359121
Unique reflections	13650
Data completeness (%)	98.9 (91.4)
Mean *I*/σ(*I*)	121.9 (61.0)
*R*-merge	0.044 (0.081)

**Table 4:** Statistics of comparative diffraction data set for a hen-egg lysozyme crystal Two data sets are obtained from an identical sample in different beam set-ups of sagittal focusing and conventional optics. The crystal size is 100 µm × 90 µm × 60 µm. Data collection conditions: oscillation range: 0 to 180° in 1° steps (180 images); exposure time: 3 s per frame. Values in parentheses refer to the highest-resolution shell (1.76–1.70 Å). *R*-merge is the residual among equivalent reflections expressed as Σ|*I* − 〈*I*〉|/Σ〈*I*〉.

	Optics setting	
	Sagittal focusing	Conventional
Space group	*P*4_3_2_1_2	*P*4_3_2_1_2
Cell constants (Å)	*a* = 78.77, *b* = 36.92	*a* = 78.87, *b* = 36.97
Wavelength (Å)	1.00	1.00
Resolution (Å)	50.0–1.70 (1.76–1.70)	50.0–1.70 (1.76–1.70)
No. of observations	176411	176133
Unique reflections	13356	13382
Data completeness (%)	99.7 (99.9)	99.9 (100.0)
Mean *I*/σ(*I*)	84.7 (34.4)	66.0 (15.1)
*R*-merge	0.036 (0.090)	0.044 (0.198)

## References

[bb1] ANSYS (2007). ANSYS Release 11.0. ANSYS Inc., Canonsburg, PA, USA.

[bb2] Bilsborrow, R. L., Atkinson, P. A., Bliss, N., Dent, A. J., Dobson, B. R. & Stephenson, P. C. (2006). *J. Synchrotron Rad.* **13**, 54–58. 10.1107/S0909049505036903 16371708

[bb3] Borsboom, M., Bras, W., Cerjak, I., Detollenaere, D., Glastra van Loon, D., Goedtkindt, P., Konijnenburg, M., Lassing, P., Levine, Y. K., Munneke, B., Oversluizen, M., van Tol, R. & Vlieg, E. (1998). *J. Synchrotron Rad.* **5**, 518–520. 10.1107/S0909049597013484 15263564

[bb4] Feng, L., Kang, L., Li, Z., Zhao, F. & Xu, C. (2008). *J. Synchrotron Rad.* **15**, 140–143. 10.1107/S0909049507066368 18296779

[bb26] Frenkel, A., Barg, B., Heald, S., Kim, K. H., Brown, F. & Stern, E. A. (1996). *Rev. Sci. Instrum.* **67**, 1–4.

[bb5] Koshelev, I., Huang, R., Graber, T., Meron, M., Muir, J. L., Lavender, W., Battaile, K., Mulichak, A. M. & Keefe, L. J. (2009). *J. Synchrotron Rad.* **16**, 647–657. 10.1107/S0909049509022353 19713639

[bb6] Kounosu, A., Iwasaki, T., Baba, S., Hayashi-Iwasaki, Y., Oshima, T. & Kumasaka, T. (2008). *Acta Cryst.* F**64**, 1146–1148. 10.1107/S1744309108035975 PMC2593688 19052371

[bb7] Kushnir, V. I., Quintana, J. P. & Georgopoulos, P. (1993). *Nucl. Instrum. Methods Phys. Res. A*, **328**, 588–591.

[bb8] Nisawa, A. *et al.* (2013). In preparation.

[bb9] Nomura, M. & Koyama, A. (1999). *J. Synchrotron Rad.* **6**, 182–184. 10.1107/S0909049598016823 15263241

[bb10] Otwinowski, Z. & Minor, W. (1997). *Methods Enzymol.* **276**, 307–326. 10.1016/S0076-6879(97)76066-X 27754618

[bb11] Quintana, V. I., Kushnir, J. P. & Rosenbaum, G. (1995). *Nucl. Instrum. Methods Phys. Res. A*, **362**, 592–594.

[bb12] Saino, H., Ukita, Y., Ago, H., Irikura, D., Nisawa, A., Ueno, G., Yamamoto, M., Kanaoka, Y., Lam, B. K., Austen, K. F. & Miyano, M. (2011). *J. Biol. Chem.* **286**, 16392–16401. 10.1074/jbc.M110.150177 PMC3091245 21454538

[bb13] Sanchez del Rio, M. & Dejus, R. J. (1997). *Proc. SPIE*, **3152**, 148–157.

[bb14] Sanchez del Rio, M. & Dejus, R. J. (1998). *Proc. SPIE*, **3448**, 340–345.

[bb15] Schulze, C., Heidenreich, G., Auderset, H., Vermeulen, D. & Freund, A. K. (1998). *Proc. SPIE*, **3448**, 156–165.

[bb16] Sparks, C. J. Jr, Borie, B. S. & Hastings, J. B. (1980). *Nucl. Instrum. Methods*, **172**, 237–242.

[bb17] Sparks, C. J. Jr, Borie, B. S. & Hastings, J. B. (1982). *Nucl. Instrum. Methods*, **195**, 73–78.

[bb18] Tanaka, T. & Kitamura, H. (2001). *J. Synchrotron Rad.* **8**, 1221–1228. 10.1107/s090904950101425x 11679776

[bb19] Ueno, G., Kanda, H., Hirose, R., Ida, K., Kumasaka, T. & Yamamoto, M. (2006). *J. Struct. Funct. Genom.* **7**, 15–22. 10.1007/s10969-005-9005-5 16645781

[bb20] Uruga, T., Tanida, H., Yoneda, Y., Takeshita, K., Goto, S. & Ishikawa, T. (2001). *Nucl. Instrum. Methods Phys. Res. A*, **467**–**468**, 782–784.

[bb21] Welnak, C., Chen, G. J. & Cerrina, F. (1994). *Nucl. Instrum. Methods Phys. Res. A*, **347**, 344–347.

[bb22] Yabashi, M., Yamazaki, H., Tamasaku, K., Goto, S., Takeshita, K., Mochizuki, T., Yoneda, Y. & Ishikawa, T. (1999). *Proc. SPIE*, **3773**, 2–13.

[bb23] Yoneda, Y., Matsumoto, N., Furukawa, Y. & Ishikawa, T. (2001). *J. Synchrotron Rad.* **8**, 18–21. 10.1107/s0909049500014515 11486491

[bb24] Yoneda, Y., Matsumoto, N., Furukawa, Y. & Ishikawa, T. (2003). *AIP Conf. Proc.* **705**, 720–723.

[bb25] Yoneda, Y., Matsumoto, N., Furukawa, Y. & Ishikawa, T. (2005). *Phys. Scr.* T**115**, 995–997.

